# Association between rheumatoid arthritis and periodontitis: a study based on a two-sample mendelian randomisation analysis

**DOI:** 10.4317/medoral.26848

**Published:** 2025-03-23

**Authors:** Qi Cai, Chen Li, Zhizen Quan, Xinyu Yang, Tong Chen, Xu Han, Xiaogang Xu

**Affiliations:** 1 Department of Stomatology, The First Affiliated Hospital of Naval Medical University, Shanghai, China

## Abstract

**Background:**

The association between Rheumatoid arthritis (RA) and Periodontitis (PD) has been increasingly recognised, yet traditional epidemiological studies face challenges in establishing associations. Therefore, this study aims to genetically assess the association between RA and PD through Mendelian randomisation (MR) analysis, using genetic variations as instrumental variables.

**Material and Methods:**

Data on RA and PD were downloaded from the EBI website. The RA data contained 8,255 cases and 409,001 controls, with a total of 24,175,266 SNPs; the chronic PD data contained 950 cases and 409,001 controls, with a total of 11,842,647 SNPs; the acute PD data contained 128 cases and 456,220 controls, with a total of 11,842,647 SNPs. Additionally, the potential association between RA and PD was investigated. The intercept between Mendelian randomisation (MR)-Egger regression, MR-PRESSO test results and funnel plots was used to analyse the horizontal pleiotropy of SNPs along with the effect of individual SNPs on inverse-variance weighting (IVW) analysis results, assessed using the leave-one-out method.

**Results:**

In total, 26 SNPs highly associated with RA were screened; MR-Egger regression (OR=1.242, 95% CI (1.032-1.494), *P*=0.031), WM (OR=1.190, 95% CI (1.015-1.395), *P*=0.032), IVW (OR=1.191, 95% CI (1.053-1.348), *P*=0.006) and weighted mode (OR=1.212, 95% CI (1.043-1.409), *P*=0.019) suggested that RA was a likelihood factor for chronic PD, whereas RA was not associated with the incidence of acute PD, and the Cochran’s Q test indicated no statistical heterogeneity between SNPs highly associated with RA. Moreover, analyses using the intercept between the MR-Egger regression, MR-PRESSO test results and funnel plot revealed no horizontal pleiotropy in SNPs highly associated with RA.

**Conclusions:**

Rheumatoid arthritis was genetically identified as a likelihood factor for PD and the onset of chronic PD, but no association was observed between RA and acute PD.

** Key words:**Periodontitis, rheumatoid arthritis, mendelian randomisation.

## Introduction

Periodontitis (PD) is a prevalent infectious disease affecting oral health globally, with up to 90% of the population impacted (1). It is characterised by specific plaque biofilms and complex host inflammatory responses, leading to tooth mobility in severe cases (2,3). Factors such as smoking, obesity, age and poor oral hygiene contribute to its progression (4-6).

Rheumatoid arthritis (RA) is a systemic autoimmune disease causing progressive joint damage. Affecting approximately 1% of the global population, RA is more common in women than in men (7). It can lead to chronic synovial hyperplasia, joint deformities and various complications if left untreated (8,9).

Recent studies have revealed significant commonalities between PD and RA in terms of risk factors, pathological features and immunological characteristics (10,11). Both conditions involve increased inflammatory cell infiltration, the release of pro-inflammatory mediators and the activation of pathways leading to bone destruction. Notably, some researchers have proposed a link between *P. *gingivalis** and the development of RA (12).

The association between RA and PD has been increasingly recognised, yet traditional epidemiological studies face challenges in establishing associations due to confounding factors and reverse causality. Although randomised controlled trials (RCTs) are considered the gold standard for inferring associations, they are often ethically constrained and difficult to implement in this context (13). Given these limitations, Mendelian randomisation (MR) analysis offers a promising alternative. This approach, often referred to as a natural RCT, allows for causal inferences while minimising the influence of confounding factors and reverse causality (14,15). Therefore, this study aims to genetically assess the association between RA and PD through MR analysis, using genetic variations as instrumental variables.

## Material and Methods

- Data Sources

In this study, both RA and PD data were downloaded from the EBI website (https://www.ebi.ac.uk/gwas/downloads/summary-statistics). The RA data (GCST90018910) contained 8,255 cases and 409,001 controls, with a total of 24,175,266 SNPs; the chronic PD data (GCST90044102) contained 950 cases and 409,001 controls, with a total of 11,842,647 SNPs; and the acute PD data (GCST90044101) contained 128 cases and 456,220 controls, with a total of 11,842,647 SNPs. The vast majority of GWAS data in this study were derived from European populations.

- Screening of Instrumental Variables

In this study, the instrumental variables (IVs) were selected first from the GWAS pooled data of exposure following a series of stringent criteria, with the specific screening steps as follows: 1) SNPs were screened using genome-wide significance levels (*P*<5×10−8) as thresholds; 2) SNPs that were palindromic sequences (i.e. A/T or G/C) were removed; palindromic sequences refer to SNPs that have the same base order on the forward strand as on the reverse strand of DNA and are in the opposite direction, making it impossible to infer whether the strand is the forward or reverse one; 3) linkage disequilibrium (LD) in SNPs was excluded for screening independent SNPs following r2 < 0.01 and genetic distance > 10,000 kb, and LD was estimated according to the European Population Genome Project reference panel; 4) F-statistics for each SNP were calculated to exclude the weak IV bias, with IVs with F < 10 defined as weak IVs and excluded from the MR analysis (16).

- Mendelian Randomisation Analysis Process

The MR analysis was performed using the TwoSampleMR package in R software (version 4.4.0), with the MRPRESSO package used for the MR-PRESS0 analysis. The specific steps were as follows: 1) MR analysis: In this study, inverse-variance weighting (IVW) (17), MR-Egger regression (18), WM (19), simple mode and weighted mode were used for analysis, and the IVW analysis results prevailed if there was no horizontal pleiotropy among the SNPs (20); 2) Statistical heterogeneity: The presence of statistical heterogeneity among SNPs was assessed using the Cochran Q test, with P≤0.05 indicating statistical heterogeneity (21); 3) Horizontal pleiotropy: The horizontal pleiotropy of SNPs was analysed using the intercept between the MR-Egger regression, MR-PRESSO test results and funnel plots, and a nonstatistically significant intercept versus 0 indicated no horizontal pleiotropy in the SNPs. Moreover, in the MR-PRESSO test, *P*>0.05, suggesting no horizontal pleiotropy in the SNPs. Additionally, the funnel plot analysis revealed symmetrical left and right distributions in SNPs, indicating no significant horizontal pleiotropy in SNPs; 4) Sensitivity analysis: The leave-one-out method was used to assess the effects of individual SNPs on the IVW analysis results, and no significant changes to the IVW analysis results after removing a single SNP indicated no significant effect of this SNP on the IVW analysis results (22). Odds ratios (ORs) were calculated using the IVW method, which combines the effects of individual SNPs on exposure and outcomes. The IVW method assumes that all genetic variants are valid IVs and that there is no horizontal pleiotropy. To address potential confounding, we used several sensitivity analyses, including MR-Egger regression, weighted median and weighted mode methods. These methods provide estimates that are robust to certain types of pleiotropy and invalid Instrumental Variables. However, it is important to note that MR inherently controls for many confounding factors by using genetic variants as IVs, as these are generally not influenced by environmental or lifestyle factors. We did not perform additional adjustments for specific confounding factors in our primary analysis, as this is not typically done in two-sample MR studies.

## Results

- Association Between Rheumatoid Arthritis and Chronic Periodontitis

Identification of Instrumental Variables: In the association analysis between RA and chronic PD, a total of 26 SNPs were finally included in the analysis after removing LD, with the F value of each SNP >10. Table 1 presents data on related SNPs, including effector alleles and effector allele frequencies.

**Figure 1:**
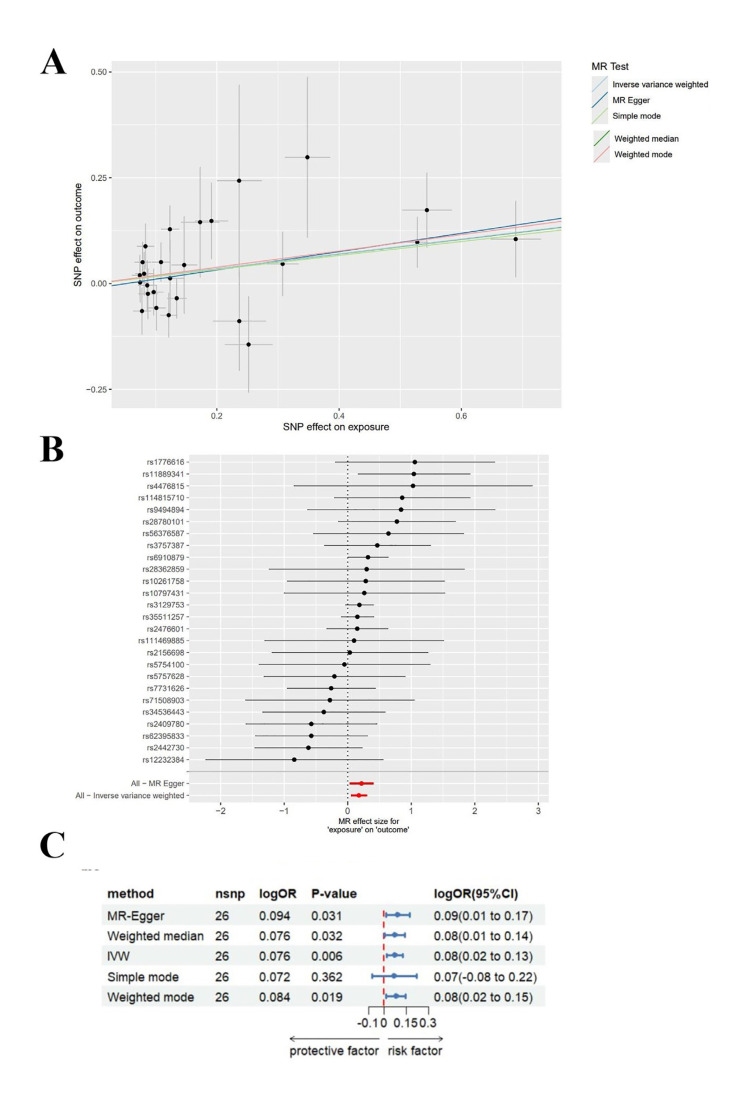
A: Forest plot of SNP-associated RA and chronic PD estimated using MR-Egger regression and IVW; B: Scatter plot of genetic association between RA and chronic PD; C: Forest plot of results from 5 methods.

Mendelian Randomisation Analysis Results: The analysis revealed no statistical significance in the results obtained using the simple mode (*P*>0.05). Moreover, MR-Egger regression (OR=1.242, 95% CI (1.032-1.494), *P*=0.031), WM (OR=1.190, 95% CI (1.015-1.395), *P*=0.032), IVW (OR=1.191, 95% CI (1.053-1.348), *P*=0.006) and weighted mode (OR=1.212, 95% CI (1.043-1.409), *P*=0.019) suggested RA as a likelihood factor for chronic PD (Fig. 1).

**Figure 2:**
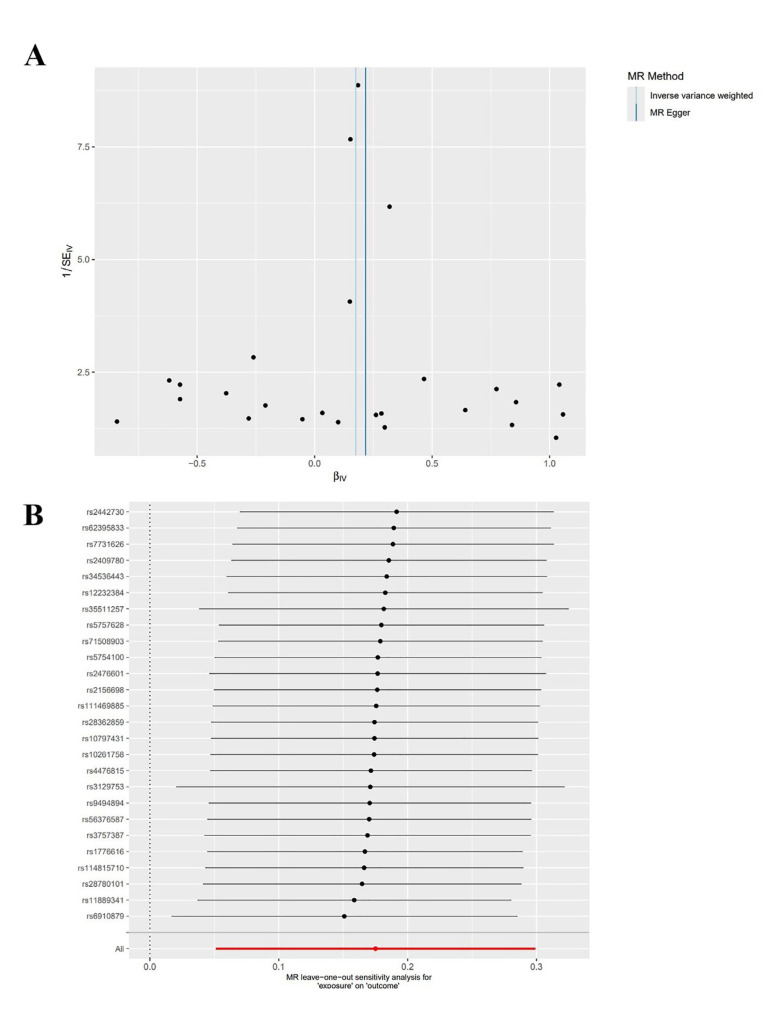
Funnel plot (A) and leave-one-out analysis plot (B) for MR analysis of RA and chronic PD.

Sensitivity Analysis: The heterogeneity test and the pleiotropy test for the MR-Egger intercept were performed on the data, indicating no heterogeneity and pleiotropy in the data (Q = 26.018, *P*=0.352; intercept = −0.011, *P*=0.554). Moreover, the funnel plot analysis revealed symmetrical left and right distributions in SNPs highly associated with RA. Furthermore, the leave-one-out analysis was performed using the remaining SNPs after the step-wise removal of a single SNP, identifying that no SNPs had a significant effect on the outcome effect (Fig. 2).

- Association Between Rheumatoid Arthritis and Acute Periodontitis

Identification of Instrumental Variables: The SNPs used in the association analysis between RA and acute PD were consistent with those of chronic PD, as shown in Table 1.

**Figure 3:**
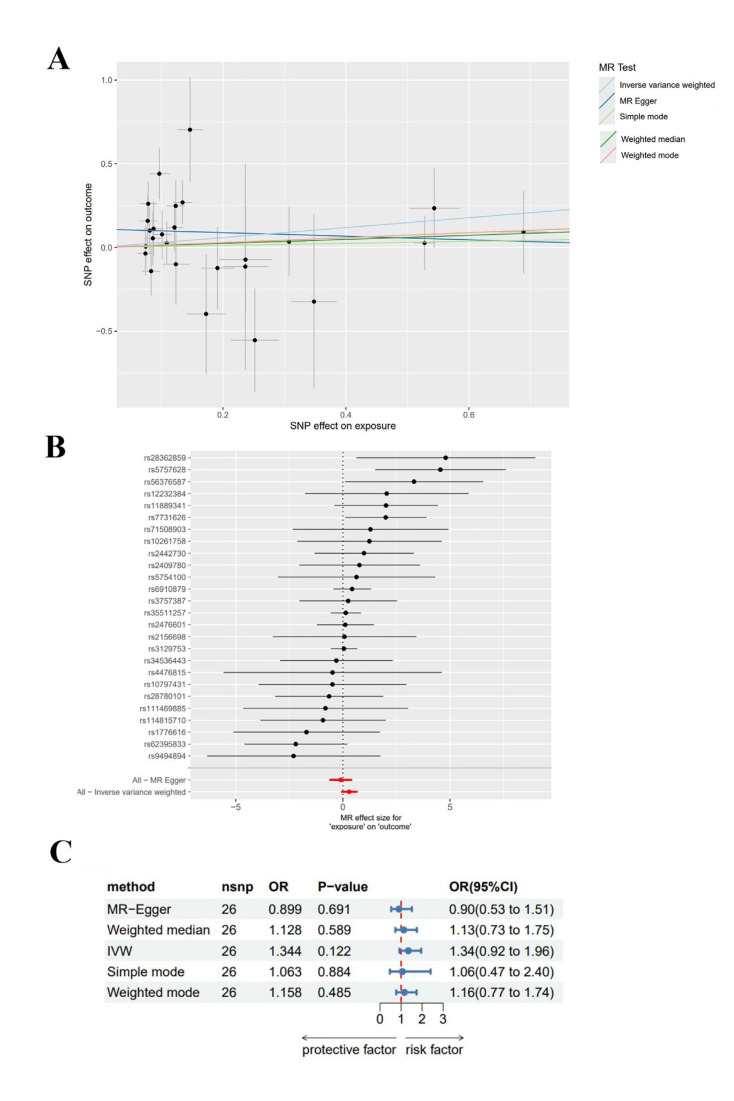
A: Forest plot of SNP-associated RA and acute PD estimated using MR-Egger regression and IVW; B: Scatter plot of genetic association between RA and acute PD; C: Forest plot of results from 5 methods.

Mendelian Randomisation Analysis Results: The analysis revealed no statistical significance in the results obtained using MR-Egger regression, WM, IVW, weighted mode or simple mode (*P*>0.05) (Fig. 3).

**Figure 4:**
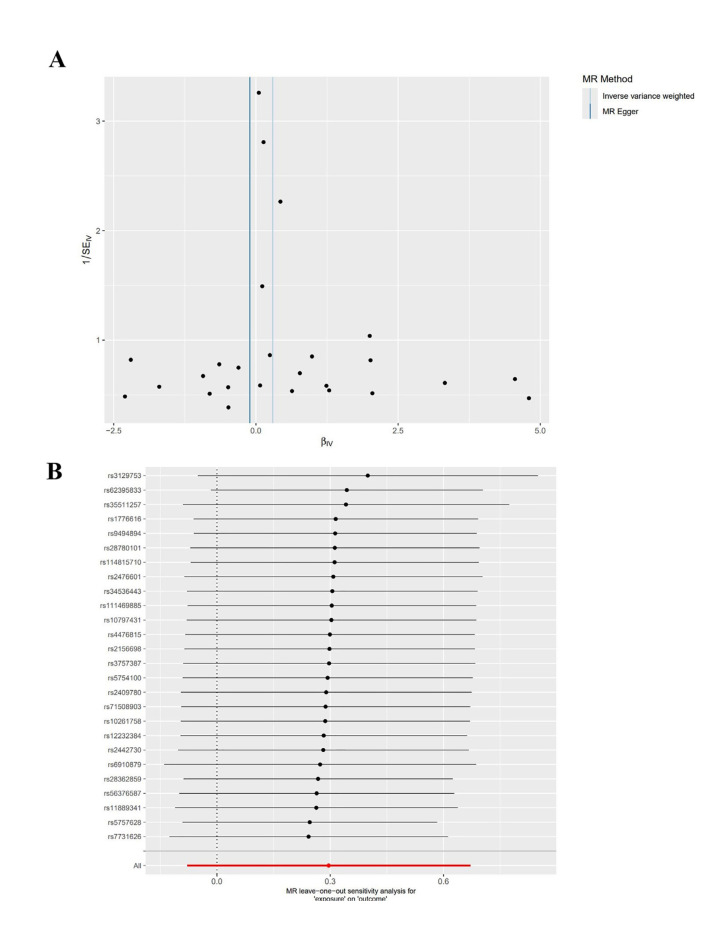
Funnel plot (A) and leave-one-out analysis plot (B) for MR analysis of RA and acute PD.

Sensitivity Analysis: The heterogeneity test and the pleiotropy test for the MR-Egger intercept were performed on the data, indicating no heterogeneity and pleiotropy in the data (Q=26.680, *P*=0.274; intercept=0.011, *P*=0.051). Additionally, the funnel plot analysis revealed symmetrical left and right distributions for SNPs highly associated with RA. Moreover, the leave-one-out analysis was performed using the remaining SNPs after the step-wise removal of a single SNP, identifying that no SNPs had a significant effect on the outcome effect (Fig. 4).

## Discussion

In this study, the association between RA and the likelihood of PD was investigated through two-sample MR analysis using large-scale GWAS pooled data, with the findings suggesting no association between RA and acute PD but indicating that RA was a likelihood factor for chronic PD (OR=1.190, 95% CI (1.015-1.395), *P*=0.032).

Many observational studies have indicated that the presence of RA may predispose individuals to PD. Kaja Eriksson *et al*. (23) concluded that smoking and age were likelihood factors for PD in both RA and control groups, without providing evidence to demonstrate that patients diagnosed with RA had a higher prevalence of PD than healthy controls. Multiple reports (24,25), including large population-based clinical studies, have revealed no association between RA and the prevalence or severity of PD. In the Nurses’ Health Study, the largest observational study so far that has investigated the timing of PD diagnosis and the number of missing teeth in female nurses (292 patients with RA, 80,840 healthy controls), no association with RA was found after follow-up for more than 12 years. However, despite the data from a population health census in Taiwan (26) suggesting that a history of PD was significantly associated with the incidence of RA, no difference in the likelihood of RA was identified in the medical records for patients with PD receiving anti-infection treatment. These reports on the relationship between RA and PD are conflicting, possibly due to a variety of factors, including ethnic differences in the study population, differences in the adjustment of confounding factors and different criteria for PD grading. Although some studies currently suggest a certain association between RA and PD, studies with sufficient sample sizes are still needed to identify the existing relationship and biological processes between RA and PD.

A large number of studies have also investigated the mechanisms between RA and PD from different aspects. In terms of genetic biomarkers, the highly polymorphic HLA-DRB1 locus (also known as the epitope-SE) is the most robust genetic factor involved in the development of both diseases (27), and people carrying the rs 2237892T allele may suffer from both diseases (28). With regard to inflammatory biomarkers, both patients with RA and PD exhibit high levels of IL1β, IL6, MMPs and TNF-α, and the increased expression of pro-inflammatory cytokines can stimulate STAT3 activation and play an essential role in the pathophysiology of RA and PD (29). As for autoantibodies, evidence suggests that the periodontal pathogens *P. *gingivalis** and *A. actinomycetemcomitans* are the two most critical microorganisms involved in the pathogenesis of RA and PD and are associated with elevated anti-cyclic citrullinated peptide antibodies, which are the diagnostic and prognostic biomarkers of RA (11,30).

In the present study, no association was observed between RA and acute PD, but RA was a likelihood factor for chronic PD. This is consistent with the findings of Larsson and Burgess (31), who conducted a comprehensive systematic review and meta-analysis using Mendelian randomization studies, demonstrating causal associations between smoking and both periodontitis and rheumatoid arthritis. However, the results of this study are inconsistent with those of Yin *et al*. (32), who investigated the association between RA and osteoarthritis and PD using the MR method, with GWAS data derived from European populations. Their results did not suggest RA and osteoarthritis as etiological factors of PD. This inconsistency in results is possibly due to differences resulting from different subgroup analyses. Specifically, the association between RA and acute/chronic PD was investigated in this study using PD as a grouping factor, whereas the study by Kang *et al*. did not divide PD into different groups, ultimately leading to divergent results.

- Limitations

This study has several limitations. First, the GWAS data used are derived from European populations; therefore, further research is needed to validate whether the experimental results could be applied to other ethnic groups. Second, the results of the two-sample MR analysis failed to group genders or identify the presence of gender-wise differences in pathogenicity. Third, as the possible biological mechanisms of RA and PD currently remain unclear, this analysis has only preliminarily identified the association between these two diseases. Finally, the MR approach cannot establish temporal relationships or causality between RA and PD but rather provides insights into their genetic association. Further research is needed to fully understand the nature and direction of this relationship.

- Strengths and Lessons Learned

Despite these limitations, the study has several strengths. The use of large-scale GWAS data and MR techniques allows us to explore genetic associations between RA and PD while minimising the influence of confounding factors. This approach provides a novel perspective on the relationship between these two conditions and generates hypotheses for future research. Our findings suggest the need for more comprehensive longitudinal studies that can track the development of both RA and PD over time. Additionally, this study highlights the value of considering genetic factors when investigating the links between systemic and oral health conditions. Future research could build on these results by combining genetic data with clinical observations to provide a more complete picture of the RA-PD relationship.

## Conclusions

In conclusion, this study genetically identified RA as a likelihood factor for the development of PD, with no association observed between RA and acute PD, but RA was identified as a likelihood factor for the onset of chronic PD.

## Figures and Tables

**Table 1:** Basic Data of SNPs in MR Analysis of RA and Acute/Chronic PD.

SNP	EA	OA	EAF	Beta	SE	P-value	F-value
rs10261758	A	G	0.711	-0.081	0.014	1.47E-08	32.08
rs10797431	T	G	0.404	-0.074	0.013	1.06E-08	32.91
rs111469885	A	G	0.096	-0.124	0.022	2.65E-08	30.95
rs114815710	A	G	0.030	-0.348	0.037	1.33E-21	90.95
rs11889341	T	C	0.250	0.123	0.015	2.71E-17	71.21
rs12232384	A	C	0.279	0.078	0.014	6.02E-08	29.37
rs1776616	G	A	0.665	0.083	0.014	2.41E-09	35.83
rs2156698	A	G	0.456	-0.075	0.013	1.44E-08	32.34
rs2409780	C	T	0.388	0.101	0.015	5.37E-12	47.21
rs2442730	A	C	0.696	-0.121	0.014	1.83E-18	77.13
rs2476601	G	A	0.887	-0.308	0.025	6.25E-34	147.82
rs28362859	C	A	0.100	0.147	0.021	9.90E-13	51.07
rs28780101	C	T	0.065	-0.191	0.027	7.66E-13	51.23
rs3129753	C	G	0.205	0.528	0.017	1.00E-200	1011.32
rs34536443	C	G	0.043	-0.237	0.043	3.03E-08	30.73
rs35511257	C	G	0.075	0.689	0.041	4.38E-64	285.10
rs3757387	C	T	0.344	0.109	0.015	1.47E-13	54.58
rs4476815	C	G	0.028	-0.236	0.036	6.92E-11	42.65
rs56376587	C	A	0.429	0.079	0.013	3.77E-09	34.84
rs5754100	C	T	0.244	0.086	0.015	9.20E-09	32.87
rs5757628	A	G	0.428	0.097	0.016	7.58E-10	37.86
rs62395833	C	G	0.032	-0.252	0.039	7.95E-11	42.37
rs6910879	G	A	0.072	0.544	0.040	7.10E-42	183.77
rs71508903	T	C	0.211	0.087	0.016	2.54E-08	30.85
rs7731626	A	G	0.279	-0.134	0.016	9.77E-18	73.17
rs9494894	C	T	0.042	0.173	0.031	2.48E-08	31.00

Note: EA: Effector allele; OA: other allele; EAF: Effector allele frequency.
